# Short‐term outcomes between robot‐assisted and open pancreaticoduodenectomy in patients with high body mass index: A propensity score matched study

**DOI:** 10.1002/cam4.6186

**Published:** 2023-05-31

**Authors:** Jingfeng Li, Lihan Qian, Yusheng Shi, Baiyong Shen, Chenghong Peng

**Affiliations:** ^1^ Department of General Surgery Pancreatic Disease Center Ruijin Hospital Shanghai Jiao Tong University School of Medicine Shanghai China; ^2^ Research Institute of Pancreatic Diseases Shanghai Jiao Tong University School of Medicine Shanghai China; ^3^ State Key Laboratory of Oncogenes and Related Genes (Shanghai) Shanghai China; ^4^ Institute of Translational Medicine Shanghai Jiao Tong University Shanghai China

**Keywords:** body mass index, overweight and obesity, pancreaticoduodenectomy, robot‐assisted, short‐term outcomes

## Abstract

**Background:**

High body mass index was considered as a risk factor for minimally invasive surgery. The short‐term outcomes of robot‐assisted pancreaticoduodenectomy (RPD) remain controversial. This study aims to investigate the feasibility and advantage of RPD in patients with high body mass index compared to open pancreaticoduodenectomy (OPD).

**Methods:**

Clinical data of 304 patients who underwent pancreaticoduodenectomy from January 2016 to December 2019 in Ruijin Hospital, Shanghai Jiao Tong University School of Medicine was collected. Patients with BMI >25 kg/m^2^ were included and divided into RPD and OPD group. After PSM at a 1:1 ratio, 75 patients of OPD and 75 patients of RPD were recorded and analyzed.

**Results:**

The RPD group showed advantages in the estimated blood loss (EBL) (323.3 mL vs. 480.7 mL, *p* = 0.010), the postoperative abdominal infection rate (24% vs. 44%, *p* = 0.010), the incidence of Clavien‐Dindo III‐V complications (14.7% vs. 28.0%, *p* = 0.042) over OPD group.

**Conclusion:**

RPD shows advantages in less EBL, lower incidence rate of Clavien‐Dindo III‐V complications over OPD in overweight and obese patients. RPD was confirmed as a safe and feasible surgical approach for overweight or obsess patients.

## 1 INTRODUCTION

Overweight and obesity are now considered nonnegligible health problems worldwide. By 2015, obesity had been diagnosed in over 107 million children and 603 million adults,^1^, ^2^ and approximately 58% of people are expected to be overweight or obese by 2030.^3^ Overweight and obesity are defined as abnormal or excessive fat accumulation that may impair health. Body mass index (BMI) is a simple index of weight‐for‐height that is commonly used to classify overweight and obesity in adults. It is defined as a person's weight in kilograms divided by the square of his height in meters (kg/m^2^). World Health Organization (WHO) defines overweight and obesity as follows: overweight is a BMI ≥25 and obesity is a BMI ≥30.^4^ The definition of obesity was BMI > 28 kg/m^2^ in China relatively. Obesity is regarded as a risk factor for some chronic diseases, such as Type 2 diabetes, hypertension, dyslipidemia, and coronary heart disease.^5^

In recent years, studies have found that obesity is an independent risk factor for the development of pancreatic and periampullary tumors.^6^, ^7^, ^8^, ^9^, ^10^ As reported in previous studies, an increase of 5 kg/m^2^ in BMI is associated with a 10% or even higher risk of developing pancreatic neoplasms.^11^, ^12^, ^13^ Based on the data of our center, the percentages of overweight and obese patients with pancreatic neoplasms increased continuously from 2016 to 2019 from 16.5% to 24.7%. More proportion of overweight and obese patients would suffer from pancreatic neoplasms due to metabolic disorders.

Pancreaticoduodenectomy is still the only radical cure for pancreatic head neoplasms and periampullary tumors.^14^ With the advancement of technology, minimally invasive pancreatic resection, especially robot‐assisted pancreaticoduodenectomy (RPD), has gained popularity. Meanwhile, it has been shown to be safe and feasible not only for benign pancreatic tumors but also for malignant tumors.^15^, ^16^, ^17^ RPD was proved with less intraoperative estimated blood loss (EBL) and fewer postoperative complications resulting in rapid recovery compared to traditional open pancreaticoduodenectomy (OPD).^17^, ^18^ However, overweight and obesity were considered to be associated with higher perioperative complications and inappropriate for pancreatic surgery. The high BMI was proved as an independent risk factor to postoperative pancreatic fistula (POPF), wound infection, and delayed gastric emptying (DGE).^19^, ^20^, ^21^ Several reasons included excessive adipose tissue, decreased operative space, and omental or mesenteric thickening^19^, ^20^, ^21^ which were supposed to explain the different short‐term outcomes between normal and overweight or obese patients. Compared to OPD, it was still unclear whether the effect of high BMI on the perioperative outcomes of RPD. This article aims to confirm the feasibility of RPD and to investigate the advantages of RPD compared to OPD for overweight and obese patients.

## 2 MATERIALS AND METHODS

### 2.1 Patient selection and surgical procedure

A total of 304 patients who underwent OPD and RPD from January 2016 to December 2019 in the Department of General Surgery, Pancreatic Disease Center, Ruijin Hospital Shanghai Jiao Tong University School of Medicine, Shanghai, China, were included in this study. This study was approved by the Ruijin Hospital Ethics Committee. Every patient was asked to sign the informed consent to make sure that they agreed with the operation and the use of data we collected before and after surgery. The study was undertaken according to the Strengthening the Reporting of Observational Studies in Epidemiology (STROBE) guidelines.^22^ The requirement for informed consent was waived due to our retrospective study. Preoperative computed tomography (CT) and magnetic resonance imaging were routinely performed for all patients. Endoscopic ultrasonography would be applied if the diagnosis of tumor remained unclear. The diagnosis of the tumor and surgical protocol was determined by our multidisciplinary team. All these surgeries were performed by the same group of surgeons in our center which had previous experience (>300 cases). All RPD were carried out by the da Vinci Si Surgical System, Intuitive TM, five‐port technique^23^ and 10‐step operative technique^24^ with experienced professors who had passed the learning curve. The inclusion criteria and exclusion criteria are as follows. 304 patients were then enrolled. There were three cases of conversion in the RPD group.

### 2.2 Inclusion criteria

1. Patients with BMI ≥25 kg/m^2^.
2. Patients underwent OPD or RPD and were pathologically diagnosed with pancreatic neoplasm located in the head of the pancreas or periampullary area.
3. The neoplasms were resectable conforming to the consensus proposed by National Comprehensive Cancer Network.

### 2.3 Exclusion criteria

1. Preoperative suspicious metastasis or unexpected involvement of major vessels were proved intraoperatively
2. History of upper‐abdomen surgery
3. Robotic‐assisted surgery converted to open laparotomy intraoperatively

**FIGURE 1:**
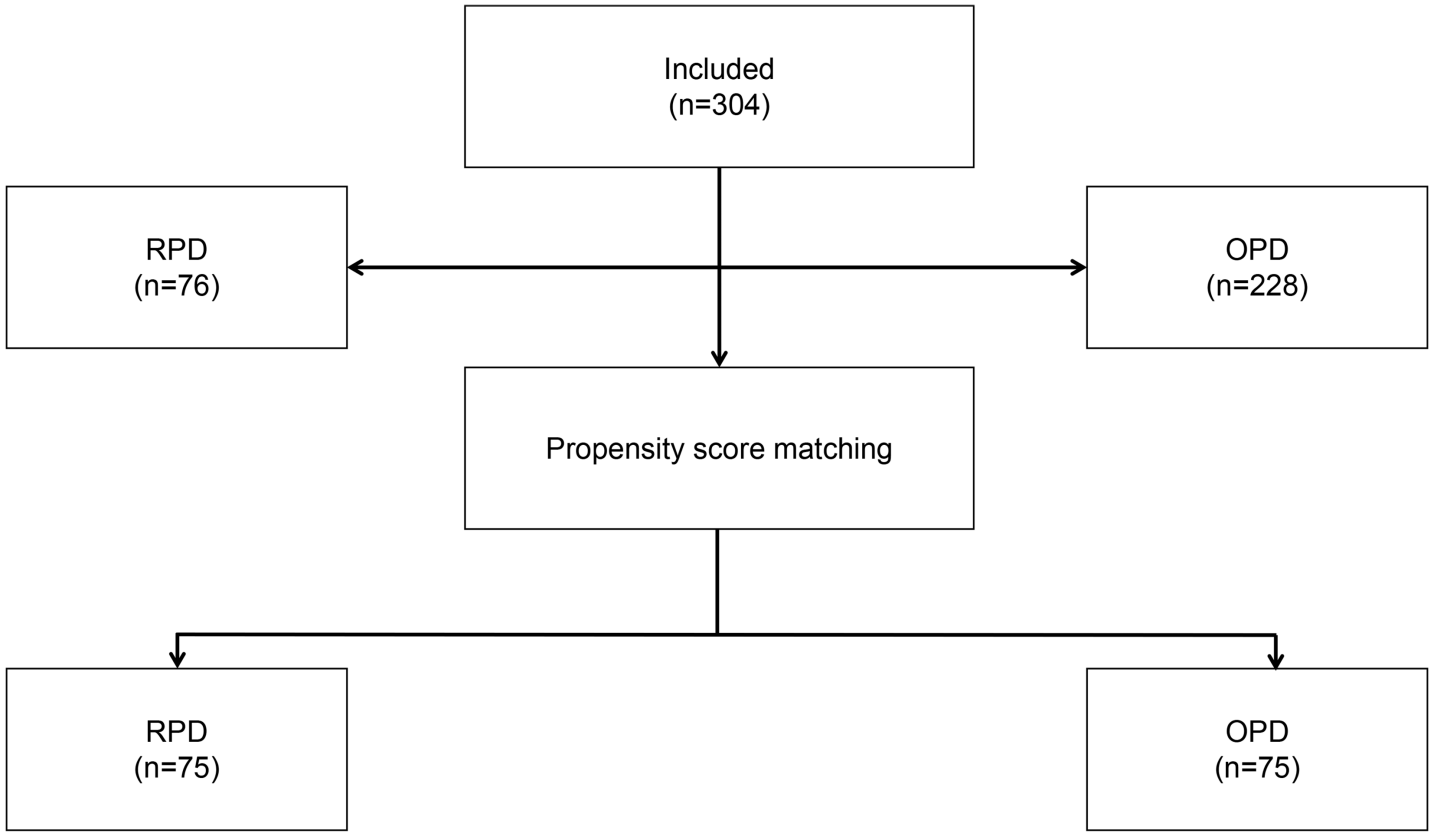
Patient selection flowchart.

The patient characteristics including age, sex, BMI, hemoglobin level, diabetes, bilirubin level, albumin level, American Society of Anesthesiologists score (ASA), alcohol/smoking history and pathological results were recorded. We used propensity score matching (PSM) to minimize the selection bias caused by different characteristics of the patients. After PSM at a 1:1 ratio, 75 pairs of patients were finally included in this study. The study flowchart is shown in Figure 1.

### 2.4 Matching and data acquisition

When comparing two groups, inevitable bias may occur due to treatment selection. Propensity score matching (PSM) is a good method to minimize the bias. As a statistical method used to process data from observational studies, it is designed to reduce the effects of selection bias and confounding variables in order to make more reasonable comparisons between experimental and control groups. We considered there was no significant difference between two groups when the value was less than 0.1. We collected patient data from our own database. The propensity score was calculated based on the covariates age, sex, BMI, ASA anesthesia score, and tumor type, and then paired one to one into two groups based on surgical type.

All outcomes were defined according to the International Study Group on Pancreatic Surgery (ISGPS).^25^ The perioperative outcomes included POPF, post‐pancreatectomy hemorrhage (PPH), bile leakage, infection, DGE, severe complications, reoperation, operation time, estimated blood loss, postoperative length of stay (LOS), and 90‐day mortality. The “Infection” is defined as postoperative signs of peritonitis, manifestations of incisional infection, the presence of positive pathogenic bacteria on culture, and imaging or surgical confirmation of the presence of infectious lesions in the abdominal cavity or under the incision, such as purulent exudate and localized abscess necrosis. The “severe complications” are defined due to Clavien‐Dindo criterion, including complications of Grade III and above, such as reoperation, abdominal gastrointestinal fistulas, effusions, and infections requiring interventional procedures, DGE requiring endoscopic treatment, and other serious life‐threatening postoperative complications and death.

Long‐term outcomes were not taken into account.

### 2.5 Statistical analysis

We used the computer software SPSS 26.0 for Windows (IBM Corp.) to do all statistical analyses. We use medians and interquartile ranges or means and standard deviations to describe continuous data, and use numbers and percentages to describe categorical data. Propensity score was calculated for each patient by using logistic regression modeling. The variables used for matching including: age, sex, BMI, Hb level, Alb level, bilirubin level, ASA score, diabetes history, smoking history, alcohol history, tumor size and pathology. Then we set a 0.01 SD as the caliper width and patients were matched 1:1. We calculated standardized mean differences before and after matching. The Mann–Whitney *U*‐test, Pearson's ∑2 test, Fisher's exact test, and Student's *t*‐test were used for data comparison. Statistical significance was defined as *p* < 0.05.

## 3 RESULTS

### 3.1 Patient characteristics

In the initial enrollment session, we enrolled a total of 228 patients undergoing OPD and 76 patients undergoing RPD. In comparing the baseline data between the two groups, we observed a significant difference in ASA scores (*p* < 0.05) between the two groups. And because of the large difference in population size between the two groups, we therefore used PSM to complete 1:1 matching. After matching, each group had 75 patients. In terms of age, sex, BMI, hemoglobin level, albumin level, bilirubin level, ASA score, tumor size, pathology results, diabetes, smoking and alcohol history, there were no significant differences between 2 groups after PSM. In the OPD group, 45 patients (60.0%) were men, the mean age was 61.5 ± 9.6 years, and the mean BMI was 27.1 ± 2.01 kg/m^2^. In the RPD group, 45 patients (60.0%) were male, the mean age was 61.8 ± 10.3 years, and the mean BMI was 27.1 ± 2.9 kg/m^2^. The patient characteristics are displayed in Table 1.

**TABLE 1:** Demographic characteristics.

	Before propensity score matching				After propensity score matching			
Characteristic	OPD (*n* = 228)	RPD (*n* = 76)	*p‐*Value	SMD	OPD (*n* = 75)	RPD (*n* = 75)	*p‐*Value	SMD
Age, yearsaa	63.4 ± 9.9	61.8 ± 10.2	0.227	15.83	61.5 ± 9.6	61.8 ± 10.3	0.870	2.61
Sex								
Male/female	135/93	46/30	0.84	2.65	30/45	30/45	1.000	0.00
BMI, kg/m^2^	27.1 ± 1.9	27.1 ± 2.97	0.865	2.05	27.1 ± 2.0	27.0 ± 2.9	0.974	0.40
25‐28	168 (73.7%)	59 (77.6%)			59 (78.7%)	58 (77.3%)		
≥28	60 (26.3%)	17 (22.4%)			16 (21.3%)	17 (22.7%)		
Normal Hb level^a^	175 (76.8%)	65 (85.5%)	0.104	22.38	61 (81.3%)	64 (85.3%)	0.511	10.74
Normal Alb level^b^	169 (74.1%)	62 (81.6%)	0.188	18.14	62 (82.7%)	61 (81.3%)	0.832	3.64
Normal bilirubin level^c^	127 (55.7%)	50 (65.8%)	0.123	20.80	47 (62.7%)	49 (65.3%)	0.734	3.64
ASA score			0.050	33.00			0.306	6.59
1	143 (62.7%)	59 (77.6%)			60 (80.0%)	58 (77.3%)		
2	74 (32.5%)	15 (19.7%)			10 (13.3%)	15 (20.0%)		
≥3	11 (4.8%)	2 (2.6%)			5 (6.7%)	2 (2.7%)		
Preoperative diabetes	54 (23.7%)	19 (25.0%)	0.816	30.30	19 (25.3%)	18 (24.0%)	0.850	3.02
History of smoking	64 (28.1%)	18 (23,7%)	0.456	10.06	20 (26.7%)	18 (24.0%)	0.707	6.21
History of alcohol	39 (17.1%)	13 (17,1%)	1.000	0.00	8 (10.7%)	13 (17.3%)	0.239	19.11
Tumor size, cm	3.02 ± 1.40	2.74 ± 1.34	0.121	20.38	3.13 ± 1.56	2.73 ± 1.35	0.104	27.35
Pathology			0.051	24.90			0.258	5.01
Benign or low‐grade malignant tumors at the pancreatic head or periampullary area^d^	42 (18.4%)	22 (28.9%)			21 (28.0%)	21 (28.0%)		
Malignant tumors at the pancreatic head^e^	125 (54.8%)	39 (51.3%)			46 (61.3%)	39 (52.0%)		
Malignant periampullary tumors^e^	61 (26.8%)	15 (19.7%)			8 (10.7%)	15 (20.0%)		

Abbreviations: Alb, albumin, ASA, American Society of Anesthesiologists; BMI, body mass index, Hb, hemoglobin, SMD, standardized mean difference.

^a^ Normal hemoglobin level is ≥11 g/dL in female and ≥ 12 g/dL in male (to convert to g/L, multiply by 10).

^b^ Normal albumin level is ≥3.5 g/dL (to convert to g/L, multiply by 10).

^c^ Normal bilirubin level is ≤34.1 μmol/L.

^d^ Lymph node clearance was not required.

^e^ Lymph node clearance was required.

### 3.2 Perioperative outcomes

There were three cases of conversion in the RPD group. One patient with BMI at 25.5 kg/m^2^ and one patient with BMI at 31.1 kg/m^2^ underwent conversion of RPD because of unexpected intense contact between the tumor margin and superior mesenteric artery (SMA)/superior mesenteric vein (SMV). One patient with BMI at 29.3 suffered intraoperative bleeding of during the resection of uncinate process of the pancreas and received OPD considering the perioperative safety. The perioperative outcomes of patients in RPD group were compared to those in OPD group and are shown in Table 2. Tche EBL was significantly lower in the RPD group than in the OPD group (323.3 vs. 480.7 mL, *p* = 0.010). The operation time and postoperative complications, including POPF, bile leakage, PPH, DGE, and reoperation, showed no differences between the 2 groups (Table 2). The occurrences of infection and severe complications (Clavien‐Dindo Grade ≥ III) were significantly higher in the OPD group than in the RPD group (44.0% vs. 24.0%, *p* = 0.010; 28.0% vs. 14.7%, *p* = 0.046). The occurrence of 90‐day mortality was lower in the RPD group (1.3% vs. 8.0%, *p* = 0.042). Six patients died within 90 days after surgery in the OPD group. The reasons included hemorrhage in two patients, severe abdominal infection in one patient, liver metastasis in one patient, and dyscrasia in the other two patients. Only one patient, who was in the RPD group, died on the postoperative day fifth because of an unexpected cardiopulmonary failure.

**TABLE 2:** Intraoperative and perioperative outcomes before and after propensity score matching.

	Before propensity score matching			After propensity score matching		
Outcome	OPD (*n* = 228)	RPD (*n* = 76)	*p* Value	OPD (*n* = 75)	RPD (*n* = 75)	*p* Value
Operation time, min	310.8 ± 66.3	301.3 ± 61.7	0.272	303.3 ± 66.0	301.3 ± 62.1	0.849
EBL, mL	411.4 (352.9)	327.0 (223.52)	0.051	480.7 (475.0)	323.3 (222.8)	0.010
R0 resection	216 (95.6%)	75 (98.7%)	0.167	70 (94.6%)	74 (98.7%)	0.209
POPF^a^	50 (21.9%)	16 (21.1%)	0.872	15 (20.0%)	16 (21.3%)	0.840
Biochemical leak	2 (4.0%)	1 (6.3%)		0 (0.0%)	1 (6.25%)	
CR‐POPF	48 (21.1%)	15 (19.7)	0.806	15 (20.0%)	15 (20.0%)	1.000
Grade B	42 (84.0%)	14 (87.4%)		13 (86.7%)	14 (87.5%)	
Grade C	6 (12.0%)	1 (6.3%)		2 (13.3%)	1 (6.25%)	
Bile leak	19 (8.3%)	3 (3.9%)	0.201	6 (8.0%)	3 (4.0%)	0.494
Abdominal infection	88 (38.6%)	18 (23.7%)	0.018	33 (44.0%)	18 (24.0%)	0.010
PPH	17 (7.5%)	2 (2.6%)	0.102	6 (8.0%)	2 (2.7%)	0.276
DGE	15 (6.6%)	6 (7.9%)	0.695	5 (6.7%)	6 (8.0%)	0.754
Complications of Grade ≥ III^b^	49 (21.5%)	11 (14.5%)	0.183	21 (28.0%)	11 (14.7%)	0.046
Reoperation	18 (7.9%)	2 (2.6%)	0.109	7 (9.3%)	2 (2.7%)	0.086
90‐day mortality	15 (6.6%)	1 (1.3%)	0.043	6 (8.0%)	1 (1.3)	0.042
Postoperative LOS^c^,d	26.5 (16.9)	24.1 (20.2)	0.317	27.4 (16.5)	24.3 (20.3)	0.292

Abbreviations: EBL Estimated blood loss, POPF postoperative pancreatic fistula, CR‐POPF clinical related postoperative pancreatic fistula, PPH post‐pancreatectomy hemorrhage, DGE delayed gastric emptying, LOS length of stay.

^a^ POPF is definded by criterion of the International Study Group of Pancreatic Fistula (ISGPF).

^b^ The classification of postoperative complications is due to Clavien‐Dindo criterion.

^c^ The cases of death were excluded in analyses of the postoperative LOS.

## 4 DISCUSSION

Obesity and overweight are thought to be associated with the development of pancreatic neoplasms and periampullary tumors.^6^, ^7^, ^8^ According to previous reports, obesity also has an impact on patients undergoing PD surgery.^19^, ^26^, ^27^ Shengliang He proposed that RPD was associated with decreased blood loss and shorter hospital stays in obese patients.^18^ Peng L indicated that the wound infection rate was lower in RPD than OPD.^17^ However, their conclusion was limited by their sample size and there were few reports or studies that compared the effects of obesity on perioperative outcomes in RPD versus OPD. Our propensity score matched study showed that the RPD group had advantages compared to the OPD group in several terms, illustrating the considerable advantage of RPD in reducing surgical trauma in obese and overweight patients.

Tjeertes EK found that obesity is a significant risk factor for postoperative abdominal infection, greater intraoperative blood loss, and a longer operation time.^27^ The possible reasons were as follows: (1) Excessive subcutaneous fat tissue predisposes these patients to impaired healing due to low regional perfusion and oxygen tension.^28^ (2) A longer operation time has been described as a significant predictor of postoperative wound infections.^29^, ^30^ (3) Impaired immunity, elevated blood glucose levels, and too much tension in the surgical incision are also factors that contribute to impaired wound healing^31^, ^32^ (4) Anatomical difficulties caused by excessive fat distribution. Meanwhile, the smaller incisions and more delicate manipulations of robot‐assisted surgery, as well as faster recovery, can bring significant advantages.^33^, ^34^

In our study, the EBL was lower in the RPD group than in the OPD group. For overweight and obese patients, the level of surgical challenge increases due to their complicated regional anatomy of the duodenum, SMA and SMV. More precise manipulation should be made because of the vascular fragility and unexpected bleeding during stretch of the mesentery. On the contrary, robot‐assisted surgical systems can filter hand tremor; thus, for obese patients with brittle tissues, a robotic approach may be a better choice than open surgery to ensure that accidental injury is avoided. Especially when dealing with vessels, robot‐assisted surgery may help prevent damaging the vascular wall and reduce the incidence of bleeding. Better field exposure and higher magnification during robot‐assisted surgery can also effectively reduce estimated blood loss. Furthermore, increased EBL was well‐documented to be associated with higher morbidity in terms of postoperative complications which could explain the lower incidence of Complications of Grade (≥ III) and abdominal infection.^35^, ^36^ We proposed that the robot‐assisted approaches can benefit the patients with less EBL and lower postoperative severe complications by more flexible and precise manipulation in overweight and obese patients.

In our study, the operation time was not different between the two groups. In previous studies and reports, robot‐assisted surgery was reported with much longer time than traditional open surgery. Reasons may include the longer preparation times needed for installation and because the procedures and processes are not as skilled as those performed during traditional surgery. Based on the previous experimental results obtained at our center with the standardized surgical procedure, ineffective intraoperative manipulations can be significantly reduced, thus achieving a stable operation time and a low level of surgical difficulty.^37^ This modular surgical concept and approach has nearly eliminated time‐consuming missteps during RPD operations, and the surgical procedure can be modified by adjusting the surgical path and sequence for overweight and obese patients individually and feasibly.

POPF represents one of the most common and serious complications. Buchs et al showed that more accurate surgical process and less manipulation around the residual pancreas in RPD could decrease the incidence of POPF.^38^ Patricio M. Polanco et al^39^ and Bing‐Yang Hu et al^40^pointed out that higher BMI was relevant with the POPF which required more precise surgical procedure. There was no significant difference of the incidence of POPF between the two groups in our study. We proposed that with the current surgical technique, RPD was safe for overweight and obese patients.

The advantages of RPD versus OPD in overweight and obese patients may be concluded as following. The operators of robot‐assisted surgery have a more comfortable operating space. During the operation, there was no need to open and close the large abdominal incision, thereby simplifying the operation procedure, reducing the level of difficulty of the exposure process, and decreasing morbidity due to infection. However, there are inevitable challenges in performing robot‐assisted surgery in overweight and obese patient population. First, thicker subcutaneous fat requires precise establishment of the Trocar orifice which is prone to slip out and affects the size of the intra‐abdominal space after pneumoperitoneum establishment. Besides, overweight and obese patients have a thicker omentum and mesentery, making them more prone to bleeding and damage to the intestinal segment during surgery when separating the transverse colon and duodenum. In addition, fatty pancreas leads to increased difficulty in suturing the pancreatic‐enteric anastomosis, which has a significant impact on the occurrence of POPF. The learning curve for RPD, especially with those overweight and obese patients requires further prospective studies to verify based on our conclusion and recent study.

There are several limitations to our study. First, there was still bias associated with patient selection and the retrospective nature of the research although PSM method was made. Second, BMI is widely used to distinguish overweight and obese people, visceral fat which was more complex to calculate by CT scan was proposed as a prior measure than BMI for clinical researches. In addition, PSM is statistically inferior to randomized studies. Further prospective randomized clinical trials should be designed and carried out to better understand the benefits of robot‐assisted surgery in overweight and obese populations.

## 5 CONCLUSION

In this study, we compared RPD surgery with OPD surgery in a population of overweight and obese patients. We found that RPD has advantages in less EBL, lower incidence rate of Clavien‐Dindo III‐V complications over OPD. RPD was confirmed as a safe and feasible surgical approach for overweight or obsess patients.

## 6 FUTURE OUTLOOK

We plan to conduct a randomized clinical trial in the future on the treatment of obese patients with robot‐assisted pancreatic surgery. The definition of obesity will not be limited to BMI, but will be defined by assessing the patient's visceral fat, subcutaneous fat, pancreatic steatosis, and other aspects. We also hope to conduct long‐term prognostic follow‐up and to provide evidence for clinical management through survival analysis.

## AUTHOR CONTRIBUTIONS

**Jingfeng Li:** Conceptualization (lead); data curation (lead); formal analysis (lead); funding acquisition (lead); investigation (lead); methodology (lead); project administration (lead); resources (lead); software (lead); supervision (lead); validation (lead); visualization (lead); writing – original draft (lead); writing – review and editing (lead). **Lihan Qian:** Conceptualization (equal); data curation (equal); formal analysis (equal); funding acquisition (equal); investigation (equal); methodology (equal); project administration (equal); resources (equal); software (equal); supervision (equal); validation (equal); visualization (equal); writing – original draft (equal); writing – review and editing (equal). **Yusheng Shi:** Conceptualization (equal); data curation (equal); formal analysis (equal); funding acquisition (equal); investigation (equal); methodology (equal); project administration (equal); resources (equal); software (equal); supervision (equal); validation (equal); visualization (equal); writing – original draft (equal); writing – review and editing (equal). **Baiyong Shen:** Conceptualization (equal); data curation (equal); formal analysis (equal); funding acquisition (equal); investigation (equal); methodology (equal); project administration (equal); resources (equal); software (equal); supervision (equal); validation (equal); visualization (equal); writing – original draft (equal); writing – review and editing (equal). **Chenghong Peng:** Conceptualization (equal); data curation (equal); formal analysis (equal); funding acquisition (equal); investigation (equal); methodology (equal); project administration (equal); resources (equal); software (equal); supervision (equal); validation (equal); visualization (equal); writing – original draft (equal); writing – review and editing (equal).

## FUNDING INFORMATION

This study was funded by the National Natural Science Foundation of China (81672325).

## ETHICS STATEMENT

This study was approved by the institutional review board of Shanghai Ruijin Hospital.

## Data Availability

The data that support the findings of this study are available from the corresponding author upon reasonable request.

## References

[1] Afshin A , Forouzanfar MH , Reitsma MB , et al. Health effects of overweight and obesity in 195 countries over 25 years. N Engl J Med. 2017;377(1):13‐27. doi:10.1056/NEJMoa1614362 28604169 PMC5477817

[2] Trends in adult body‐mass index in 200 countries from 1975 to 2014: a pooled analysis of 1698 population‐based measurement studies with 19.2 million participants. Lancet. 2016;387(10026):1377‐1396. doi:10.1016/S0140-6736(16)30054-X 27115820 PMC7615134

[3] Brawer R , Brisbon N , Plumb J . Obesity and cancer. Prim Care. 2009;36(3):509‐531. doi:10.1016/j.pop.2009.04.005 19616153

[4] Bracci PM . Obesity and pancreatic cancer: overview of epidemiologic evidence and biologic mechanisms. Mol Carcinog. 2012;51(1):53‐63. doi:10.1002/mc.20778 22162231 PMC3348117

[5] Haslam DW , James WP . Obesity. Lancet. 2005;366(9492):1197‐1209. doi:10.1016/S0140-6736(05)67483-1 16198769

[6] Zheng W , McLaughlin JK , Gridley G , et al. A cohort study of smoking, alcohol consumption, and dietary factors for pancreatic cancer (United States). Cancer Causes Control. 1993;4(5):477‐482. doi:10.1007/BF00050867 8218880

[7] Levi Z , Rottenberg Y , Twig G , et al. Adolescent overweight and obesity and the risk for pancreatic cancer among men and women: a nationwide study of 1.79 million Israeli adolescents. Cancer. 2019;125(1):118‐126. doi:10.1002/cncr.31764 30417331

[8] Arslan AA . Anthropometric measures, body mass index, and pancreatic cancer. Arch Intern Med. 2010;170(9):791‐802. doi:10.1001/archinternmed.2010.63 20458087 PMC2920035

[9] Genkinger JM , Spiegelman D , Anderson KE , et al. A pooled analysis of 14 cohort studies of anthropometric factors and pancreatic cancer risk. Int J Cancer. 2011;129(7):1708‐1717. doi:10.1002/ijc.25794 21105029 PMC3073156

[10] Jiao L , Berrington De Gonzalez A , Hartge P , et al. Body mass index, effect modifiers, and risk of pancreatic cancer: a pooled study of seven prospective cohorts. Cancer cause Control. 2010;21(8):1305‐1314. doi:10.1007/s10552-010-9558-x PMC2904431 20383573

[11] Berrington De Gonzalez A , Sweetland S , Spencer E . A meta‐analysis of obesity and the risk of pancreatic cancer. *Brit* . J Cancer. 2003;89(3):519‐523. doi:10.1038/sj.bjc.6601140 PMC2394383 12888824

[12] Larsson SC , Orsini N , Wolk A . Body mass index and pancreatic cancer risk: a meta‐analysis of prospective studies. Int J Cancer. 2007;120(9):1993‐1998. doi:10.1002/ijc.22535 17266034

[13] Renehan AG , Tyson M , Egger M , Heller RF , Zwahlen M . Body‐mass index and incidence of cancer: a systematic review and meta‐analysis of prospective observational studies. Lancet. 2008;371(9612):569‐578. doi:10.1016/S0140-6736(08)60269-X 18280327

[14] Cameron JL , He J . Two thousand consecutive pancreaticoduodenectomies. J Am Coll Surg. 2015;220(4):530‐536. doi:10.1016/j.jamcollsurg.2014.12.031 25724606

[15] Correa‐Gallego C , Dinkelspiel HE , Sulimanoff I , et al. Minimally‐invasive vs open pancreaticoduodenectomy: systematic review and meta‐analysis. J Am Coll Surg. 2014;218(1):129‐139. doi:10.1016/j.jamcollsurg.2013.09.005 24275074

[16] Nigri G , Petrucciani N , La Torre M , et al. Duodenopancreatectomy: open or minimally invasive approach? Surgeon. 2014;12(4):227‐234. doi:10.1016/j.surge.2014.01.006 24525404

[17] Peng L , Lin S , Li Y , Xiao W . Systematic review and meta‐analysis of robotic versus open pancreaticoduodenectomy. Surg Endosc. 2017;31(8):3085‐3097. doi:10.1007/s00464-016-5371-2 27928665

[18] He S , Ding D , Wright MJ , et al. The impact of high body mass index on patients undergoing robotic pancreatectomy: a propensity matched analysis. Surgery. 2020;167(3):556‐559. doi:10.1016/j.surg.2019.11.002 31837833

[19] Del Chiaro M . Impact of body mass index for patients undergoing pancreaticoduodenectomy. World J Gastrointest Pathophysiol. 2013;4(2):37‐42. doi:10.4291/wjgp.v4.i2.37 23755369 PMC3676538

[20] Mathur A , Luberice K , Paul H , Franka C , Rosemurgy A . Increasing body mass index portends abbreviated survival following pancreatoduodenectomy for pancreatic adenocarcinoma. Am J Surg. 2015;209(6):969‐973. doi:10.1016/j.amjsurg.2014.12.037 25916614

[21] You L , Zhao W , Hong X , et al. The effect of body mass index on surgical outcomes in patients undergoing pancreatic resection: a systematic review and meta‐analysis. Pancreas. 2016;45(6):796‐805. doi:10.1097/MPA.0000000000000525 27295531

[22] von Elm E , Altman DG , Egger M , Pocock SJ , Gøtzsche PC , Vandenbroucke JP . The strengthening the reporting of observational studies in epidemiology (STROBE) statement: guidelines for reporting observational studies. Lancet. 2007;370(9596):1453‐1457. doi:10.1016/S0140-6736(07)61602-X 18064739

[23] Galvez D , Sorber R , Javed AA , He J . Technical considerations for the fully robotic pancreaticoduodenectomy. J Vis Surg. 2017;3:81. doi:10.21037/jovs.2017.05.08 29078644 PMC5638600

[24] Shi Y , Wang W , Qiu W , et al. Learning curve from 450 cases of robot‐assisted Pancreaticoduocectomy in a high‐volume pancreatic center. Ann Surg. 2021;274(6):e1277‐e1283. doi:10.1097/SLA.0000000000003664 31651533

[25] Bassi C , Marchegiani G , Dervenis C , et al. The 2016 update of the international study group (ISGPS) definition and grading of postoperative pancreatic fistula: 11 years after. Surgery. 2017;161(3):584‐591. doi:10.1016/j.surg.2016.11.014 28040257

[26] Dindo D , Muller MK , Weber M , Clavien PA . Obesity in general elective surgery. Lancet. 2003;361(9374):2032‐2035. doi:10.1016/S0140-6736(03)13640-9 12814714

[27] Tjeertes EEKM , Hoeks SSE , Beks SSBJ , et al. Obesity‐‐a risk factor for postoperative complications in general surgery? BMC Anesthesiol. 2015;15(1):112. doi:10.1186/s12871-015-0096-7 26228844 PMC4520073

[28] Hopf HW , Hunt TK , West JM , et al. Wound tissue oxygen tension predicts the risk of wound infection in surgical patients. Arch Surg. 1997;132(9):997‐1004, 1005‐1004; discussion 1005. doi:10.1001/archsurg.1997.01430330063010 9301613

[29] Mullen JT , Davenport DL , Hutter MM , et al. Impact of body mass index on perioperative outcomes in patients undergoing major intra‐abdominal cancer surgery. Ann Surg Oncol. 2008;15(8):2164‐2172. doi:10.1245/s10434-008-9990-2 18548313

[30] Kurmann A , Vorburger SA , Candinas D , Beldi G . Operation time and body mass index are significant risk factors for surgical site infection in laparoscopic sigmoid resection: a multicenter study. Surg Endosc. 2011;25(11):3531‐3534. doi:10.1007/s00464-011-1753-7 21638185

[31] Tanaka S , Inoue S , Isoda F , et al. Impaired immunity in obesity: suppressed but reversible lymphocyte responsiveness. Int J Obes Relat Metab Disord. 1993;17(11):631‐636. 8281221

[32] Stryker LS , Abdel MP , Morrey ME , Morrow MM , Kor DJ , Morrey BF . Elevated postoperative blood glucose and preoperative hemoglobin A1C are associated with increased wound complications following total joint arthroplasty. J Bone Joint Surg Am. 2013;95(9):808‐814. doi:10.2106/JBJS.L.00494 23636187

[33] Lai ECH , Yang GPC , Tang CN . Robot‐assisted laparoscopic pancreaticoduodenectomy versus open pancreaticoduodenectomy‐‐a comparative study. Int J Surg. 2012;10(9):475‐479. doi:10.1016/j.ijsu.2012.06.003 22732431

[34] Zhou N , Chen J , Liu Q , et al. Outcomes of pancreatoduodenectomy with robotic surgery versus open surgery. Int J Med Robot. 2011;7(2):131‐137. doi:10.1002/rcs.380 21412963

[35] Ball CG , Pitt HA , Kilbane ME , Dixon E , Sutherland FR , Lillemoe KD . Peri‐operative blood transfusion and operative time are quality indicators for pancreatoduodenectomy. HPB (Oxford). 2010;12(7):465‐471. doi:10.1111/j.1477-2574.2010.00209.x 20815855 PMC3030755

[36] Li S , Zhou K , Lai Y , Shen C , Wu Y , Che G . Estimated intraoperative blood loss correlates with postoperative cardiopulmonary complications and length of stay in patients undergoing video‐assisted thoracoscopic lung cancer lobectomy: a retrospective cohort study. BMC Surg. 2018;18(1):29. doi:10.1186/s12893-018-0360-0 29792183 PMC5966911

[37] Shi Y , Jin J , Qiu W , et al. Short‐term outcomes after robot‐assisted vs open pancreaticoduodenectomy after the learning curve. JAMA Surg. 2020;155(5):389‐394. doi:10.1001/jamasurg.2020.0021 32129815 PMC7057168

[38] Buchs NC , Addeo P , Bianco FM , Ayloo S , Benedetti E , Giulianotti PC . Robotic versus open pancreaticoduodenectomy: a comparative study at a single institution. World J Surg. 2011;35(12):2739‐2746. doi:10.1007/s00268-011-1276-3 21947494

[39] Polanco PM , Zenati MS , Hogg ME , et al. An analysis of risk factors for pancreatic fistula after robotic pancreaticoduodenectomy: outcomes from a consecutive series of standardized pancreatic reconstructions. Surg Endosc. 2016;30(4):1523‐1529. doi:10.1007/s00464-015-4366-8 26139506

[40] Hu B , Wan T , Zhang W , et al. Risk factors for postoperative pancreatic fistula: analysis of 539 successive cases of pancreaticoduodenectomy. World J Gastroenterol. 2016;22(34):7797‐7805. doi:10.3748/wjg.v22.i34.7797 27678363 PMC5016380

